# Effectiveness of Graphene Oxide (GO) in Activating the Mitochondrial Pathway of Oxidative Stress-Induced Apoptosis in Breast Cancer Cells

**DOI:** 10.3390/cells14211717

**Published:** 2025-11-01

**Authors:** Rafał Krętowski, Beata Szynaka, Małgorzata Borzym-Kluczyk, Natalia Tyszka, Agata Jabłońska-Trypuć, Maciej Gil, Marzanna Cechowska-Pasko

**Affiliations:** 1 Department of Pharmaceutical Biochemistry, Medical University of Bialystok, Mickiewicza 2A, 15-222 Bialystok, Poland; malgorzata.borzym-kluczyk@umb.edu.pl (M.B.-K.); natalia.tyszka@umb.edu.pl (N.T.); marzanna.cechowska-pasko@umb.edu.pl (M.C.-P.); 2 Department of Histology and Embryology, Medical University of Białystok, Waszyngtona 13, 15-269 Białystok, Poland; beataszynaka@gmail.com; 3 Department of Chemistry, Biology and Biotechnology, Bialystok University of Technology, Wiejska 45A, 15-351 Bialystok, Poland; a.jablonska@pb.edu.pl (A.J.-T.); mgil290502@gmail.com (M.G.)

**Keywords:** apoptosis, breast cancer, cytotoxicity, oxidative stress, graphene oxide (GO)

## Abstract

**Highlights:**

**What are the main findings?**

- Graphene oxide induces oxidative stress and apoptosis in MDA-MB-231 but not in ZR-75-1 cell lines;
- Apoptosis is induced by a caspase-9-dependent mitochondrial pathway.

**What are the implications of the main findings?**

- Insights into the mechanism of graphene oxide action on breast cancer MDA-MB-231 cells are provided;
- Graphene oxide opens up new possibilities in the fight against breast cancer.

**Abstract:**

Due to its unique physicochemical properties, graphene oxide (GO) is used in nanomedicine. Many studies have examined the effects of GO on cancer cells. However, there are no data on the mechanisms of action of GO in breast cancer. The aim of this study was to analyze the cytotoxic effect and mechanisms of action of GO on MDA-MB-231 and ZR-75-1 cell lines. Our findings show that GO induced cytotoxicity in MDA-MB-231 but not in ZR-75-1 cells. The cytotoxic effect of GO on fibroblasts was negligible. Cytotoxicity was associated with ROS synthesis, decreased mitochondrial membrane potential, and apoptosis/necrosis in MDA-MB-231 cells. In addition, we observed cell cycle arrest and increased P21 protein expression in MDA-MB-231 cells. Furthermore, we observed increased levels of proapoptotic proteins, decreased levels of antiapoptotic proteins, and activation of caspase-9 and caspase-3/7 in MDA-MB-231 cells. This study elucidates the possible mechanisms of action of GO in breast cancer cells.

## 1. Introduction

Breast cancer (BC) is one of the most common malignancies, surpassing lung cancer. Despite advances in early diagnosis and treatment, BC remains a serious health and epidemiological problem worldwide [1]. Traditional therapeutic methods, such as surgery, radiotherapy, and chemotherapy, fail to deliver the desired results while generating a number of side effects, such as lymphedema, damage to normal tissue, and tumor recurrence [2]. A better understanding of the molecular basis of BC is expected to provide better therapeutic options [1].

Much of our knowledge about BC is based on in vitro studies conducted on various breast cancer cells, including ZR-75-1 and MDA-MB-231. ZR-75-1 is a cell line that retains its epithelial phenotype, while MDA-MB-231 does not express the epithelial marker but exhibits high levels of vimentin [3]. Innovative nanotechnology treatment methods using graphene oxide (GO) offer an alternative therapeutic avenue for breast cancer, which has previously demonstrated significant resistance to standard treatments [2].

Graphene-based nanomaterials (GBMs) are widely used in nanomedicine; for example, in cancer therapy, diagnostics, bio-imaging, drug carriers, biosensors, and antibacterial agents [4,5]. GO is a novel two-dimensional carbon nanosheet that, compared to pure graphene, is characterized by a larger specific surface area and hydrophilic groups, such as hydroxyl, carboxyl, and epoxy groups. It is also characterized by high stability in aqueous solutions [5], and its cytotoxicity depends on the particulate state, the number of layers, surface groups, and size and shape [6]. In addition, GO cytotoxicity is an important factor in bionanomedical applications [6]. GO can interact with membrane lipids and receptors, disrupt cellular metabolism, and induce oxidative stress or programmed cell death (PCD) [4]. PCD is a genetically controlled cell death process that disrupts cell homeostasis. Apoptosis is characterized by numerous morphological and biochemical changes in the cell. Morphological changes include cell shrinkage, nuclear condensation/fragmentation, and the formation of apoptotic bodies, while biochemical changes are accompanied by the activation of numerous enzymatic proteins, including caspases [4].

Recently, some reports have demonstrated the immediate cytotoxic effect of GO on different cancer cells [7]. Gurunathan et al. suggested that GO inhibits the viability and proliferation of ovarian cancer cells [7]. Shen et al. found that GO inhibits colorectal tumor growth both in vitro and in vivo [8]. Moreover, Wand et al. indicated that GO inhibits the migration and invasion of cervical cancer cells [9]. Szczepaniak et al. indicated that reduced graphene oxide (rGO) induces apoptosis in glioblastoma multiforme cell lines U87 and U118 [10].

In our previous studies, we found that rGO induced cytotoxicity, reduced proliferation, enhanced oxidative stress, and increased apoptosis in MDA-MB-231 and ZR-75-1 cells [11]. Krętowski et al. demonstrated that rGO treatment resulted in cell cycle arrest, accompanied by deregulated P21 protein expression in breast cancer cell lines [12]. The same study also indicated that rGO disrupts mitochondrial membrane potential and induces apoptosis and autophagy in MDA-MB-231 and ZR-75-1 cells [12].

The aim of the study was to evaluate the cytotoxic effect of GO on MDA-MB-231 and ZR-75-1 breast cancer cell lines and human skin fibroblasts (CRL-1474). Furthermore, we investigated the effects of GO on the oxidative stress, apoptosis/necrosis, expression of proapoptotic and antiapoptotic proteins, and changes in the ultrastructure of breast cancer cells.

We examined the percentage of apoptotic/necrotic cells and the mechanism of apoptosis (active caspase-9 and -3/7, mitochondrial membrane potential, and the ROS level) using flow cytometry. We validated the results regarding the percentage of apoptotic/necrotic cells using fluorescence microscopy and Western immunoblot techniques. Furthermore, we examined the ultrastructure of MDA-MB-231 cells using electron microscopy.

After a thorough analysis of many publications, the authors hypothesized that the induction of the cytotoxic effect of GO on MDA-MB-231 cells may be related to an increase in intracellular ROS levels and the induction of apoptosis.

## 2. Materials and Methods

### 2.1. Reagents

Cell culture: L-15 Medium (1×) + GlutaMAX^TM^-I, trypsin-EDTA, medium RPMI 1640, penicillin, streptomycin, and fetal bovine serum Gold (FBS Gold) were ordered from Gibco (San Diego, CA, USA), while 3-(4,5-dimethylthiazol-2-yl)-2,5-diphenyltetrazolium bromide was provided by Merck (St. Louis, MO, USA).

Flow cytometry methods: The FITC Annexin V apoptosis detection Kit I and the JC-1 MitoScreen Kit by BD Pharmingen^TM^ (San Diego, CA, USA) were used. The FAM-LEHD-FMK Caspase-9 Assay, FAM-DEVD-FMK Caspase-3/7 Assay, and Intracellular Total ROS Activity Assay were purchased from ImmunoChemistry Technologies (Davis, CA, USA).

Western blot analysis: Monoclonal (rabbit) antibody: anti-human P21, anti-human Bcl-xl, anti-human P-Bim, anti-human Cyt c, anti-human Noxa, polyclonal (rabbit) anti-human β-tublin antibody, HRP conjugated with anti-human secondary antibody against rabbit IgG, ECL Reagent, RIPA buffer, and Protease/Phosphatase Inhibitor Cocktail were purchased from Cell Signaling (St. Louis, MO, USA). The BCA Protein Assay Kit was obtained from Thermo Scientific (Rockford, IL, USA). Immuno-Blot PVDF Membranes for Protein Blotting were obtained from Bio-Rad (Hercules, CA, USA).

Other reagents: Graphene oxide was provided by AGP (Zielona Góra, Poland). N-acetylcysteine was provided by Merck (St. Louis, MO, USA).

### 2.2. Cell Cultures and GO—Treatment

Breast cancer cell lines ZR-75-1, and MDA-MB-231 were provided by the American Type Culture Collection (ATCC) and cultured with the use of L-15 Medium (1×) + GlutaMAX^TM^-I (MDA-MB-231 cells), or in RPMI Medium 1640 (1×) + GlutaMAX^TM^-I (ZR-75-1 cells), while non-cancerous human skin fibroblasts (CRL-1474) were cultured in DMEM (1×) + GlutaMAX^TM^-I. The medium contained 10% heat-inactivated fetal bovine serum GOLD (GIBCO, NY, USA) and 1% penicillin/streptomycin antibiotic (GIBCO, NY, USA). Cell culture was conducted with the use of Falcon flasks (BD Pharmingen^TM^, San Diego, CA, USA) in the Galaxy S+ CO_2_ incubator (RS Biotech, Irvine, UK), at 37 °C and 5% CO_2_ (or no CO_2_ for MDA-MB-231 cells), with 95% or 100% air. At the confluence reaching approximately 70%, the cells were treated with 0.05% trypsin and 0.02% EDTA in order to detach them and counted in a Scepter Cell Counter (Millipore, MA, USA). At a density of about 2.5 × 10^5^ per well, cells were seeded in 2 mL of an appropriate medium in six-well plates. Subsequently, after 24 h of incubation, cell growth media were replaced with fresh ones containing different concentrations of GO suspensions (10 μg/mL to 300 μg/mL) to explore the influence of GO on breast cancer cells (MDA-MB-231 and ZR-75-1). The cell lines without GO constituted the negative controls. Subsequently, the cells were incubated for 48 h and retained for further analyses.

### 2.3. MTT Assay

Cell viability assay was conducted according to Carmichael et al. [13]. Breast cancer cells (MDA-MB-231 and ZR-75-1) and human skin fibroblasts (CRL-1474), were seeded in 24-well plates (2.5 × 10^4^ per well). Next, the cells were tested and analyzed as previously described [14,15,16,17].

### 2.4. Detection of Apoptosis and Necrosis

The apoptosis/necrosis of breast cancer MDA-MB-231 and ZR-75-1 cell lines were measured by flow cytometry on the FACSCanto II cytometer (BD, San Diego, CA, USA). Both types of breast cancer cells (2.0 × 10^5^ per well) were cultured in 2 mL of medium in six-well plates. After 24 h, the medium was replaced with GO (50 μg/mL). The breast cancer cell lines were exposed to studied compounds for 48 h. An Annexin V-FITC. Next, the cells were tested and analyzed as previously described [12,18].

### 2.5. Fluorescent Microscopy

The breast MDA-MB-231 and ZR-75-1 cancer cell lines were stained with fluorescent dyes, acridine orange (AO) and ethidium bromide (EB), was used for the evaluation of the apoptotic/necrotic cells. The breast cancer cells were incubated on cover glass with GO (50 μg/mL) for 48 h. After these times, the cells were incubated according as described in our previous publication [12].

### 2.6. JC-I Assay

Disruption of mitochondrial membrane potential (∆Ψm) was identified using the cationic fluorescence dye JC-I through flow cytometry. Breast cancer cell lines: MDA-MB-231 and ZR-75-1 were cultured in 2 mL of L-15 medium or RPMI medium, in 6-well plates (2.0 × 10^5^ cells per well). The media were removed and replaced with fresh media containing GO (50 μg/mL) after 24 h. Subsequently, the cells were incubated for 48 h. The examined cells were incubated according to the method described previously in our publication [12].

### 2.7. Measurement of Caspase 3/7 and 9 Activity

Caspase-9 and caspase-3/7 activity was measured using the FAM-LEHD-FMK or FAM-DEVD-FMK (ImmunoChemistry Technologies). For 48 h, the MDA-MB-231 and ZR-75-1 cells were cultured with GO (50 µg/mL). Measurement of caspase 9 and 3/7 activity was marked according to the method published earlier [12,19].

### 2.8. Western Blot Analysis and Chemiluminescence Detection

The MDA-MB-231 was incubated (50 μg/mL of GO) for 48 h. The breast cancer cells were lysed with RIPA buffer with addition a protease/phosphatase-inhibitor cocktail. Next, the samples of lysates were prepared as previously our described [12].

Protein lysates were separated using SDS-PAGE gel, and the proteins were transferred onto PVDF membranes. The next steps were carried out as described in a previous report [12]. The membranes were then probed overnight at 4 °C with the following antibodies at the indicated concentrations: monoclonal (rabbit) anti-human P21 Waf1/Cip1 antibody (1:1000), monoclonal (rabbit) anti-human Bcl-xl antibody (1:1000), monoclonal (rabbit) anti-human P-Bim antibody (1:1000), monoclonal (rabbit) anti-human Cyt c antibody (1:1000), monoclonal (rabbit) anti-human Noxa antibody (1:1000), and polyclonal (rabbit) anti-human β-tubulin antibody (1:1000). After incubation, with HRP conjugated with anti-human secondary antibody against rabbit IgG at 1:1000, the tested separated proteins were identified using ECL Reagent (Cell Signaling).

### 2.9. Total Protein Content in Cells

The proteins of cell lysates were measured using the BCA Protein Assay Kit [20].

### 2.10. Cell Cycle Analysis

The distribution of the cell cycle phases was studied with the use of flow cytometry. The MDA-MB-231 and ZR-75-1 cells were cultured in 6-well plates (2.0 × 10^5^ per well) and exposed to 50 μg/mL of GO for 48 h. At the end of the treatment, the cells were prepared as described in the previous publication [12,21].

### 2.11. Transmission Electron Microscopy

The breast cancer cells were incubated in L-15 medium in six-well plates. After 24 h of incubation, the medium was removed and replaced with a suspension of GO (50 μg/mL). MDA-MB-231 cells were then incubated for 48 h. After incubation, the cells were centrifuged (1000× *g*, 5 min), fixed in a mixture of 2.5% glutaraldehyde and 2% paraformaldehyde in 0.1 M cacodylate buffer (CB) at pH 7.0 and 4 °C for 1 h, and then agar was added and cut into small blocks (1 × 1 mm). The blocks with embedded cells were washed three times for 15 min each in CB at 4 °C, incubated for 1 h in 1% osmium tetroxide in CB at 4 °C for 1 h, then rinsed with distilled water and dehydrated in solutions of increasing concentrations of ethanol and propylene oxide. The sections were embedded in glycidyl ether 100 (Epon 812 SERVA). Ultrathin sections were cut, placed on nickel or copper grids, contrasted with uranyl acetate and lead citrate, and examined in an OPTON 900 transmission electron microscope (Zeiss, Oberkochen, Germany). The procedure was carried out as described in our previous publication [22].

### 2.12. Total Intracellular ROS Synthesis

The level of total ROS was determined using the Intracellular Total ROS Activity Assay (ImmunoChemistry Technologies, Davis, CA, USA) was measured with flow cytometry (BD FACS Canto II, San Diego, CA, USA) using typical green-emission-channel optics (530/30 nm). The MDA-MB-231 and ZR-75-1 cell lines at a density of 2.0 × 10^5^ cells were cultured in DMEM or RPMI medium. After 24 h, the medium was replaced with GO (50 μg/mL). For 48 h the two breast cancer cell lines were incubated. Subsequently, the cells were detached (10^6^ cells per mL), and the pelleted cells were suspended in medium and centrifuged at 200× *g*. Then, the breast cancer cell pellet was resuspended in 1× Assay Buffer. Total ROS Green reagent (10 µL) was added to each 490 µL sample. The samples were incubated for 1 h at 37 °C in incubator (5% CO_2_). In a separate ROS experiment, cells were preincubated (for 1 h) with 5 mmol/L of NAC.

### 2.13. Statistical Analysis

Differences between treatments and untreated examined cells were analyzed using one-way ANOVA, followed by Dunnett’s or Tukey’s tests. Data were presented as means ± SD from three independent measurements.

## 3. Results

### 3.1. GO Cytotoxicity in Fibroblasts and Breast Cancer Cells

**Figure 1:**
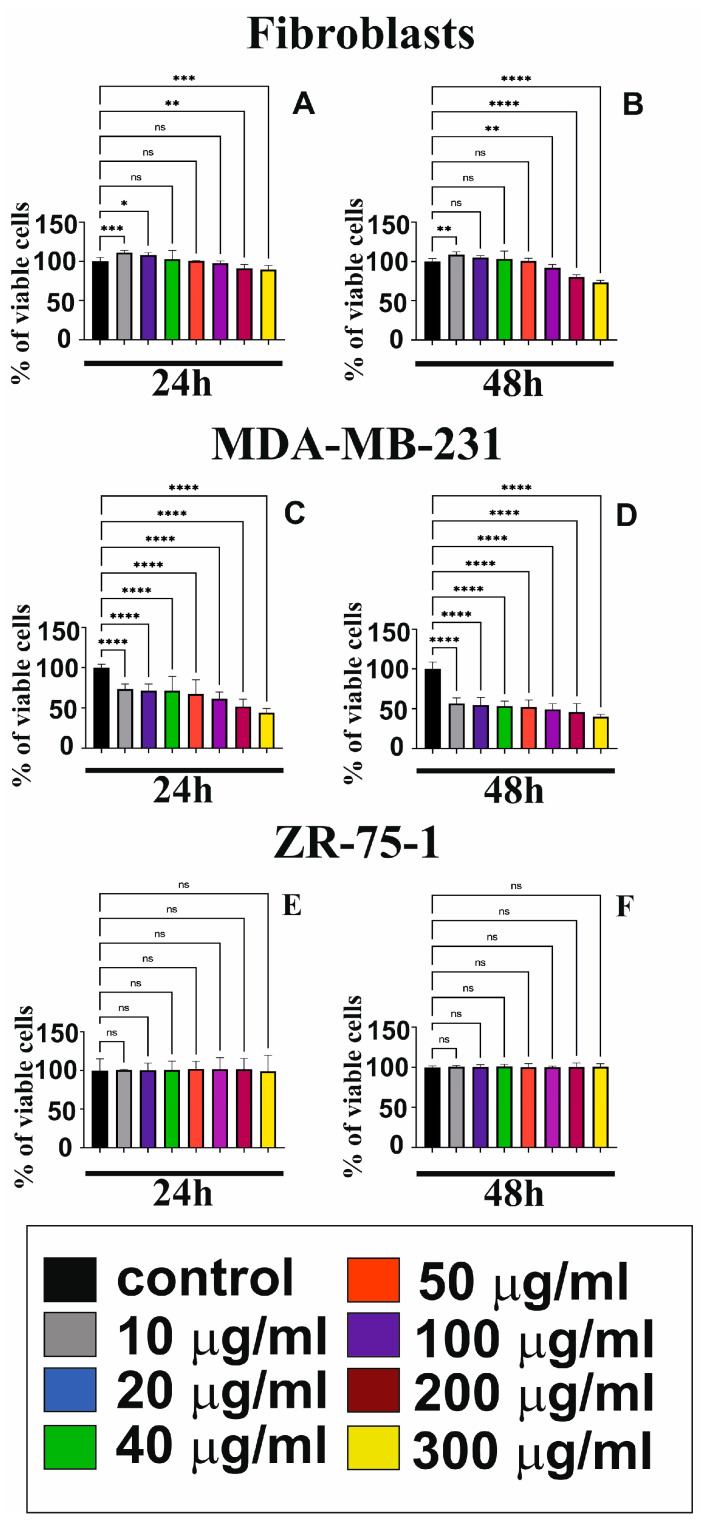
The cytotoxicity effects of GO (10 to 300 μg/mL) on human skin fibroblasts (**A**,**B**), MDA-MB-231 (**C**,**D**), and ZR-75-1 (**E**,**F**) for different treatment periods (24 h and 48 h). Mean values from three independent experiments ± SD are presented. Significant alterations are expressed relative to the controls and marked with asterisks. * *p* = 0.0197, ** *p* = 0.004, *** *p* = 0.0003, **** *p* < 0.0001, ns—not statistically significant.

The cytotoxic effects of GO at different concentrations (10 to 300 μg/mL) on fibroblasts (Figure 1A,B) and the MDA-MB-231 (Figure 1C,D) and ZR-75-1 (Figure 1E,F) cell lines were tested with an MTT assay for 24 h and 48 h. Our study shows that GO reduced the cell viability of MDA-MB-231 and fibroblasts but not ZR-75-1. As a result of incubation of cells with GO, the viability of MDA-MB-231 cells decreased depending on the dose and exposure time. (Figure 1C,D). Additionally, the cytotoxic effect on the viability of these cells was significantly more noticeable after incubation with higher concentrations of GO. In contrast to the ZR-75-1 cell lines, the MDA-MB-231 cells were sensitive to different concentrations of GO (Figure 1E,F). In normal skin fibroblasts (CRL-1474) (Figure 1A,B), we observed that a low concentration of GO induced cell viability, while higher concentrations of GO (above 100 μg/mL) reduced fibroblast viability in a time- and dose-dependent manner. It is worth noting that the cytotoxic effects of GO on the fibroblasts were negligible compared to those on the MDA-MB-231 cells. The MDA-MB-231 cell line proved to be the most sensitive as compared to other cells tested.

### 3.2. GO Influence on Apoptosis and Necrosis and Apoptosis Markers of Breast Cancer Cells

**Figure 2:**
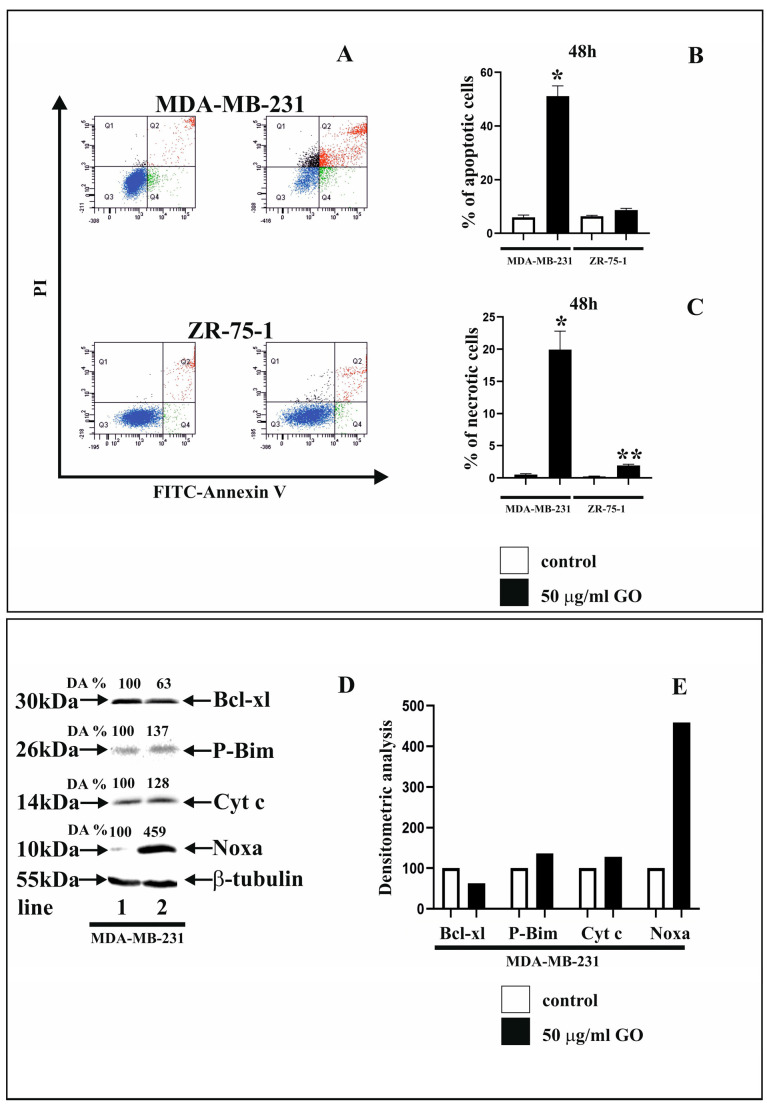
The influence of GO on apoptosis (**A**,**B**) and necrosis (**A**,**C**) in MDA-MB-231 and ZR-75-1 cells evaluated with the use of the Annexin V-FITC Apoptosis Detection Kit. The cells were treated with 50 μg/mL of GO for 48 h. Next, the cells were subjected to double staining with the use of FITC-Annexin V and PI. Representative dot plots for Annexin V-FITC/PI staining are presented (**A**–**C**): quadrant 1, necrotic cells: An−/Pi+; quadrant Q2, late apoptotic cells: An+/PI+; quadrant Q3, living cells: An−/PI−; quadrant Q4, early apoptotic cells: An+/PI−. The percentage of apoptotic cells was the sum of the percentage of quadrants Q4 and Q2. (**D**,**E**) present the Western immunoblot and densitometric analysis (DA) of Bcl-xl, P-Bim, Cyt-c, and Noxa expression in MDA-MB-231 cell lines treated with 50 μg/mL of GO (line 2), as compared to the control (line 1) for 48 h. A representative Western immunoblot is shown. Densitometric analysis (DA) is presented as a relative fold change compared to untreated cells, where the expression level was set as 100. Panel (**E**) shows a graph of densitometric analysis of the expression of the studied proteins. The expression of β-tubulin served as a control for protein loading. Mean values from three independent experiments ± SD are presented. Significant alterations are expressed relative to adequate controls and marked with asterisks (* *p* = 0.00018; ** *p* = 0.00021).

Flow cytometry (FACSCanto II (BD, San Diego, CA, USA)) analysis was used to assess apoptosis and necrosis in both MDA-MB-231 (Figure 2A,B) and ZR-75-1 (Figure 2A,C) cells. According to the obtained results, GO at a concentration of 50 μg/mL stimulated apoptosis in the MDA-MB-231 cell line after 48 h of incubation; however, we did not observe any effect in ZR-75-1 cells. Representative dot plots for MDA-MB-231 and ZR-75-1 cells obtained from FACS analysis using Annexin V-FITC/PI staining are presented in Figure 2. Furthermore, our results clearly indicate that treatment with GO at the 50 μg/mL concentration for 48 h significantly increased necrosis compared to the control (Figure 2A,C) in both cell lines tested. A particularly strong necrotic response was observed in the MDA-MB-231 cell line.

The effect of GO treatment on apoptosis was validated by analyzing the expression of apoptotic markers using Western immunoblot analysis. Because GO did not demonstrate pro-apoptotic activity in the ZR-75-1 cell line, protein expression studies were performed only for MDA-MB-231 cells. Figure 2D presents the results of Western immunoblot analysis of apoptotic marker expression in MDA-MB-231 cells treated with 50 μg/mL of GO for 48 h. The expressions of pro-apoptotic proteins (P-Bim, Cyt c, Noxa) and the anti-apoptotic protein Bcl-xl were analyzed. The obtained results indicate an inhibitory effect of GO on Bcl-xl protein expression (Figure 2D, line 2) compared to the control, untreated cells (Figure 2D, line 1). However, the expressions of P-Bim, Cyt c, and Noxa proteins under the influence of applied GO increased in the MDA-MB-231 cells (Figure 2D, line 2) compared to the control cells (Figure 2D, line 1). Furthermore, densitometric analysis was performed, and calculations for the expressions of Bcl-xl, P-Bim, Cyt c, and Noxa were performed (Figure 2D,E).

### 3.3. The Effect of GO on Nuclear Morphology of Breast Cancer Cells

**Figure 3:**
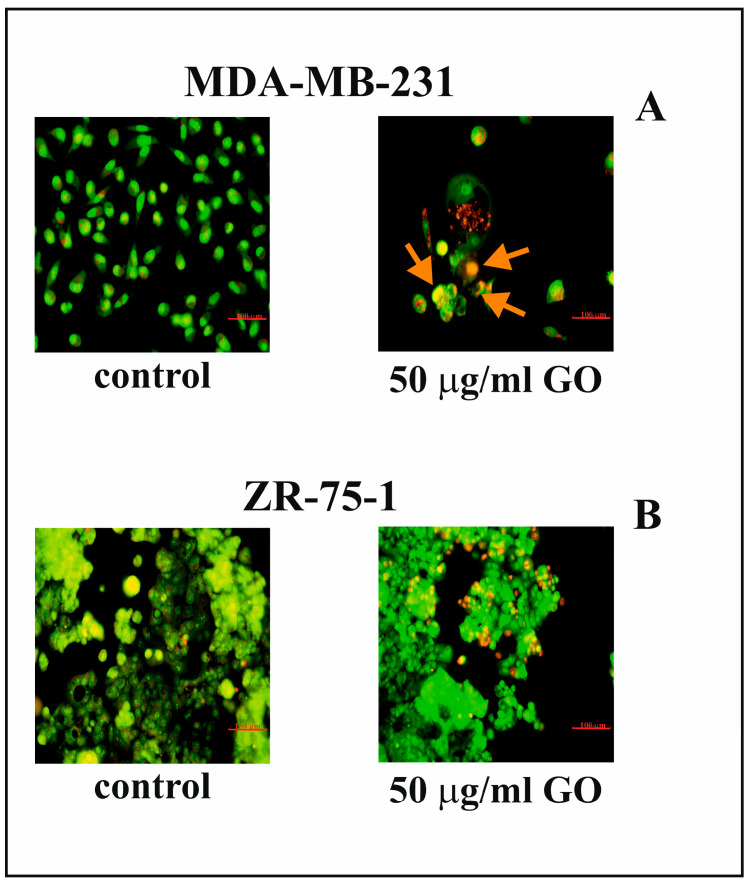
The influence of GO on apoptosis in MDA-MB-231 (**A**) and ZR-75-1 (**B**) cell lines, analyzed using fluorescence microscopy. The cells were subjected to GO (50 µg/mL) for 48 h and stained with AO/EB. The cells were imaged using fluorescence microscopy at 200-fold magnification and analyzed in order to identify living and apoptotic cells. Orange arrows indicate apoptotic cells. Representative images from one of three independent experiments are shown. Scale bar: 100 μm.

Fluorescence microscopy analysis was applied to estimate apoptotic cell morphology (Figure 3A,B). MDA-MB-231 and ZR-75-1 cells were exposed to 50 µg/mL of GO for 48 h. GO treatment induced apoptosis in MDA-MB-231 cells; however, it did not cause similar changes in the ZR-75-1 cell line (Figure 3A,B). Differences were identified between the nuclei of GO-treated and control MDA-MB-231 cells. Incubation of MDA-MB-231 cells with GO for 48 h resulted in nuclei shrinkage and the condensation and fragmentation of marginalized chromatin in apoptotic cells compared to control cells. The presence of apoptotic bodies and cell shrinkage was also confirmed.

### 3.4. The Effect of GO on Mitochondrial Membrane Potential (∆Ψm)

**Figure 4:**
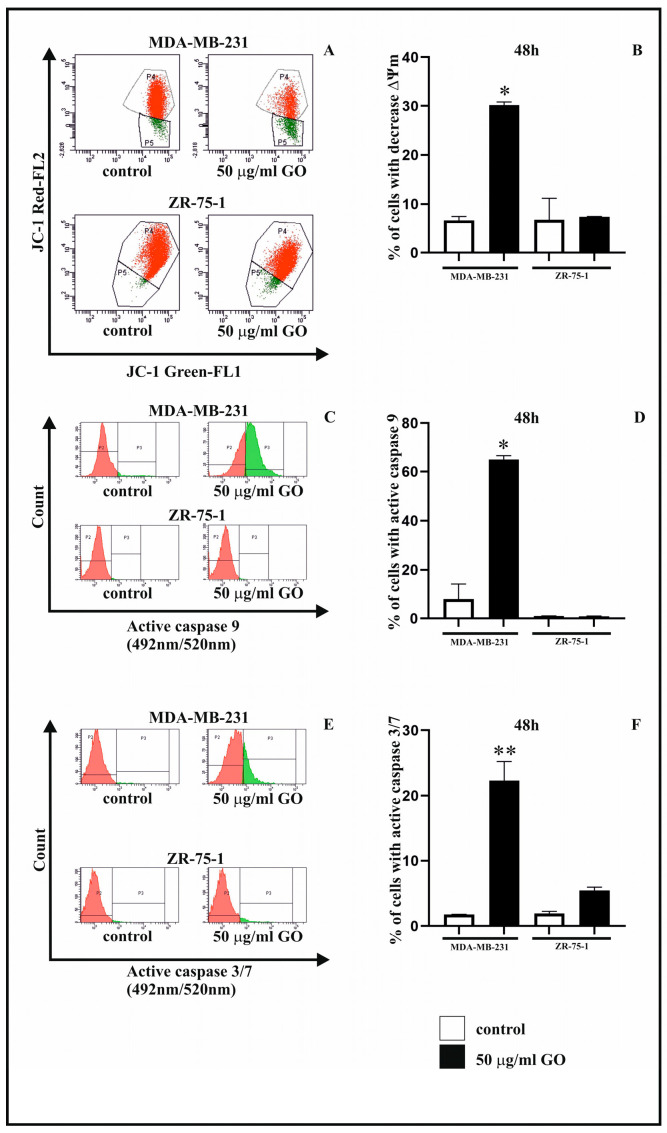
The influence of GO on mitochondrial membrane potential and activation of caspase-9 and -3/7 in the MDA-MB-231 and ZR-75-1 cell lines after exposure to 50 μg/mL of GO for 48 h. Panel (**A**) shows the flow cytometry analysis results of ΔΨ_m_ in the breast cancer cells. The *X*-axis and *Y*-axis represent green and red fluorescence, respectively. Gate P2—populations of cells with normal ΔΨm; gate P3—population of cells with decreased ΔΨ_m_. The right panel (**B**) shows the percentage of MDA-MB-231 and ZR-75-1 cells with decreased ΔΨm. Panels (**C**–**F**) show the flow cytometry analysis results of active caspase-9 and caspase-3/7 in MDA-MB-231 and ZR-75-1 cells. Panels (**C**,**E**) show representative histograms of MDA-MB-231 and ZR-75-1 cells stained with FAM-LEHD-FMK caspase-9 and FAM-DEVD-FMK caspase-3/7, respectively. Panels (**D**,**F**) show the percentage of MDA-MB-231 and ZR-75-1 cells with active caspase-9 and caspase-3/7, respectively. Gate P2—populations of cells without active caspase-9 or caspase-3/7; gate P3—populations of cells with active caspase-9 or caspase-3/7. The right panels (**D**,**F**) show the percentage of MDA-MB-231 and ZR-75-1 cells with active caspase-9 or caspase-3/7. Significant alterations are expressed relative to the controls and marked with asterisks. * *p* = 0.00029; ** *p* = 0.00047.

Figure 4 A,B show the changes induced by GO in mitochondrial membrane potential (ΔΨm) in MDA-MB-231 and ZR-75-1 cells. A reduction in mitochondrial membrane potential was confirmed for the MDA-MB cell line, but not for the ZR-75-1 cell line. MDA-MB-231 cells exposed to GO at a concentration of 50 μg/mL showed an approximately four-fold decrease in ΔΨm compared to the untreated control.

In the present study, the potential effects of 48 h of incubation with GO on the activation of caspase-9 (Figure 4C,D) and caspase-3/7 (Figure 4E,F) were investigated. An increase in active caspase-9 (over 60%) and active caspase-3/7 (over 20%) after exposure to GO at a concentration of 50 µg/mL was noted only in the MDA-MB-231 cell line compared to control cells.

### 3.5. The GO Induces G2/M Phase Arrest in MDA-MB-231 Cell Lines

**Figure 5:**
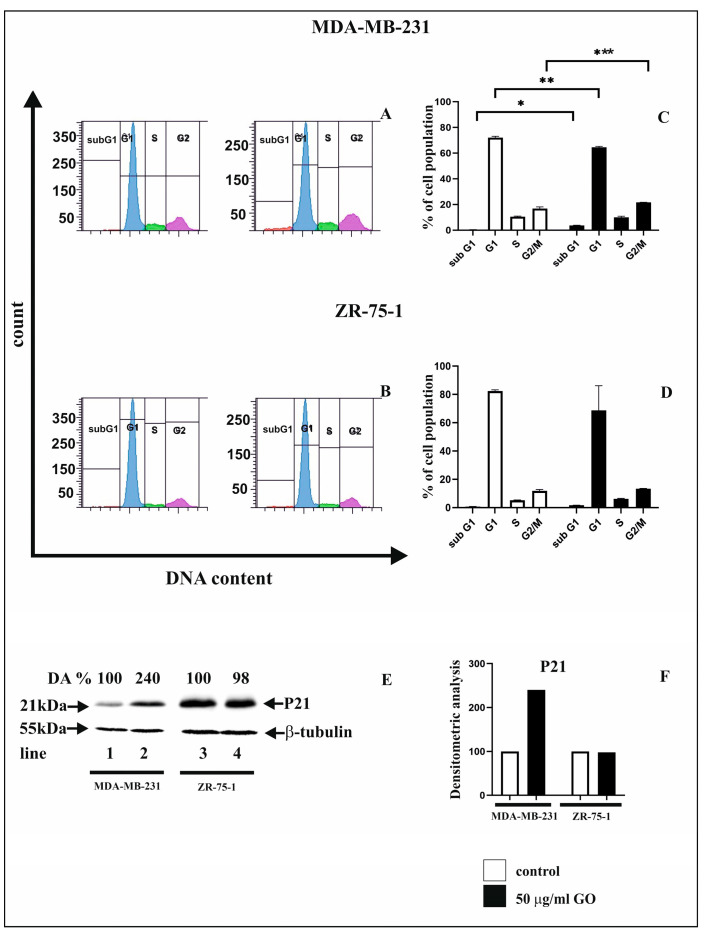
Flow cytometric analysis results of cell cycle in MDA-MB-231 (**A**,**C**) and ZR-75-1 (**B**,**D**) cells exposed to 50 µg/mL of GO for 48 h. Histogram representation of the cell cycle profiles obtained from flow cytometry measurements (**A**,**B**), and a bar graph presenting the percentage of cell cycle distribution in MDA-MB-231 (**C**) and ZR-75-1 (**D**) cells. Western blot and densitometric analysis results of P21 protein expression in MDA-MB-231 and ZR-75-1 (**E**) cells incubated with 50 µg/mL GO for 48 h. Samples containing 15 μg of protein were submitted to electrophoresis and immunoblotting. Densitometric analysis (DA) is presented as a relative fold change in comparison to untreated controls, the expression level of which was set as 100. (**F**) presents a graph of the densitometric analysis of the expressions of the studied proteins. The expression of β-tubulin served as a control for protein loading. Mean values of flow cytometry analysis from three independent experiments ± SD are presented. Significant alterations are expressed relative to controls and marked with asterisks. * *p* = 0.00029; ** *p* = 0.00073; *** *p* = 0.0033.

Figure 5 shows the flow cytometric analysis results of the cell cycle in MDA-MB-231 and ZR-75-1 cells treated with 50 µg/mL GO for 48 h. The effects of GO (50 µg/mL) on specific cell cycle phases (subG1, G1, S, G2/M) were analyzed in MDA-MB-231 and ZR-75-1 cells using flow cytometry. This study allowed us to quantify the percentage of cells in different cell cycle phases (Figure 5A–D). The percentage of cells in the G1 phase was lower compared to untreated control cells. Furthermore, cell cycle arrest in the G2/M phase and induction of the subG1 phase were observed, which correlates with the induction of apoptosis in MDA-MB-231 cells. This effect was not observed in ZR-75-1 cells. In contrast, after 48 h of incubation in GO, fewer cells remained in the G1 and induced subG1 phases compared to control MDA-MB-231 cells.

To verify these results, we performed Western immunoblot analysis of the P21 protein, which is involved in inhibiting cell cycle progression (Figure 5E). Increased P21 protein expression was observed in MDA-MB-231 cells exposed to 50 µg/mL GO (Figure 5E). We then performed DA and quantified P21 expression (Figure 5F).

### 3.6. The Morphological Changes in MDA-MB-231 Cells Exposed to GO

**Figure 6:**
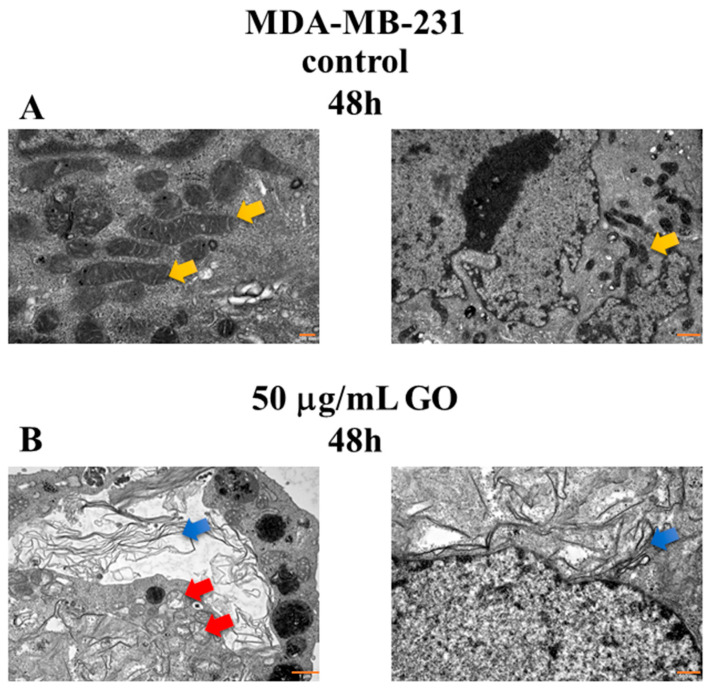
TEM analysis results of the interaction of GO with MDA-MB-231 cells. Morphological changes appeared in the MDA-MB-231 cells. The MDA-MB-231 cell lines were incubated with 50 μg/mL GO for 48 h. Control cells (**A**): The cytoplasm contained small oval or rod-shaped mitochondria with a medium-electron-dense matrix (yellow arrow). Treatment cells (**B**): Mitochondria varied in size and shape and were slightly larger than in the control group. They often exhibited focal swelling and destruction of mitochondrial cristae, or segmental and sometimes complete effacement of the limiting membranes and/or mitochondrial cristae (red arrow). Some cells had mitochondria with a thickened matrix, but in some cases, segmental effacement of the limiting membranes was also observed. Blue arrows: GO fibrils. Magnification ×50,000 and ×15,000 (Panel (**A**)), magnification ×15,000 and ×30,000 (Panel (**B**)). Scale bar: 200 nm or 1 μm (Panel (**A**)), and 1 μm or 500 nm (Panel (**B**)).

Figure 6 shows the morphological changes in the ultrastructure of breast cancer cell line MDA-MB-231 incubated with or without GO (50 μg/mL) for 48 h. Figure 6A shows the morphology of control MDA-MB-231 cells (panel A). The cells were oval in shape with irregular outlines and numerous fine surface projections. The nuclei were large and irregular, with deep invaginations, dispersed chromatin, and a very thin rim of heterochromatin at the periphery. Large, extensive nucleoli with both fibrillar and granular material were usually visible. The cytoplasm contained small oval or rod-shaped mitochondria with a medium-electron-dense matrix, sparse, short RER channels, usually single Golgi apparatuses, and numerous polyribosomes in the cytoplasm. Lysosomes were occasionally seen.

The MDA-MB-231 cells treated with GO (50 μg/mL) for 48 h were irregularly shaped, with surface projections and numerous deep invaginations (panel B). Numerous electron-dense GO fibrils were visible near the cells, on the surface of the cell membranes, and within the invaginations. GO fibrils were also present within the cytoplasm. Small clusters were sometimes surrounded by small sections of the membrane and sometimes in contact with the cytoplasm. The cell nuclei showed numerous deep invaginations, with heterochromatin clumped at the periphery, but also in small clusters throughout the nuclei. In many cells, segmental blurring of the nuclear membranes was visible, especially when GO fibrils were nearby. The nucleoli were large and exhibited intense, uniform density. Mitochondria varied in size and shape and were slightly larger than in the control group. They often exhibited focal swelling and destruction of mitochondrial cristae, or segmental and sometimes complete effacement of the limiting membranes and/or mitochondrial cristae. Some cells had mitochondria with a thickened matrix, but in some cases, segmental effacement of the limiting membranes was also observed. Numerous lysosomes and sparse polyribosomes were present in the cytoplasm. In addition, necrotic and disintegrating cells were observed, as well as cells with apoptotic changes.

### 3.7. The Effect of GO on ROS Synthesis in Breast Cancer Cell Lines

**Figure 7:**
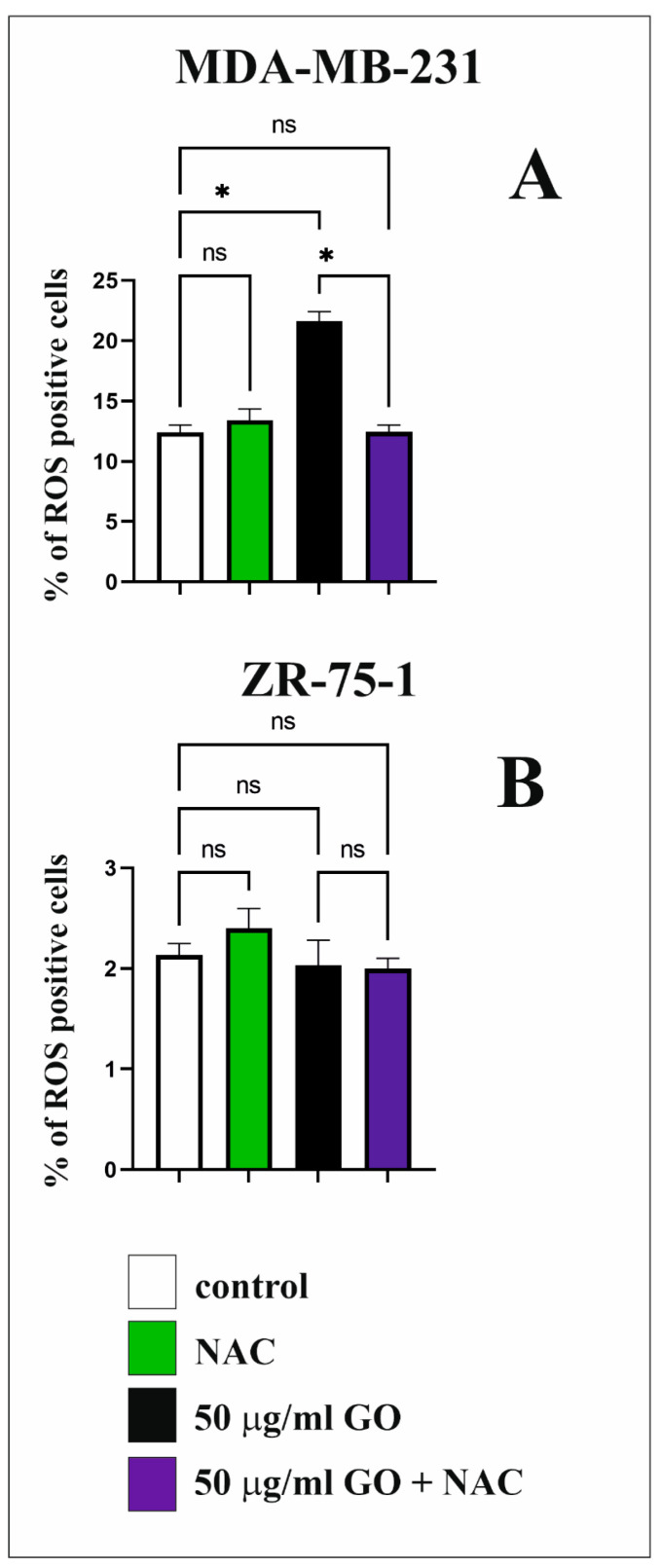
ROS production induced by GO in MDA-MB-231 (**A**) and ZR-75-1 cells (**B**). The breast cancer cells were incubated with 50 μg/mL of GO for 48 h. Significant differences compared to the adequate control group. * *p* < 0.0001, ns—not statistically significant.

Figure 7 shows the effects of 50 μg/mL GO, NAC, and GO + NAC on intracellular ROS synthesis (Figure 7A,B) in both breast cancer cell lines. The intracellular level of total ROS level was examined using the flow cytometry method by measuring fluorescence with a ROS assay kit. The breast cancer cell lines were exposed to 50 μg/mL GO for 48 h. Incubating MDA-MB-231 cells with GO induced ROS increase in these cells compared to control cells. Preincubation of MDA-MB-231 cells with 50 μg/mL GO + NAC resulted in a significant decline in the intracellular ROS levels as compared to the cells incubated with 50 μg/mL GO alone. We did not observe this effect in ZR-75-1 cells.

## 4. Discussion

Breast cancer is one of the most common cancers in women, and traditional treatment methods do not provide satisfactory therapeutic effects. Therefore, modern methods for treating breast cancer are being explored. The use of nanomedicine, including GO, is an innovative method for the treatment of breast cancer.

The MDA-MB-231 and ZR-75-1 cell lines are widely used in breast cancer experimental modeling. ZR-75-1 cells, classified as luminal molecular subtype A, exhibit a morphology characterized by mass aggregates. Luminal breast cancer cells are characterized by positive ER and/or PR expression. MDA-MB-231 cells, classified as a basic molecular subtype, exhibit a stellate aggregate morphology. In addition, MDA-MB-231 cells are ER-, PR-, and E-cadherin-negative and express mutant p53 [11].

Many studies have indicated the cytotoxic effect of GO on bacterial, normal, and cancer cells. Some studies have indicated that GO may enhance the viability of some human cancer cells [23]. This contradictory effect is related to the formation of a blood protein corona around the GO. In our study, we observed a slight enhancement of fibroblast viability at low doses of GO (10 μg/mL). Higher doses (100 μg/mL and above) of GO exhibited cytotoxic effects on normal human skin fibroblasts. Ruiz et al. indicated that GO can enhance cell adhesion and proliferation [23]. The oxide groups in GO can improve cell growth. Various studies have shown that GO has antitumor effects on osteosarcoma, cervical cancer, and other tumor cells [8], while the role and mechanism of GO in BC are still unclear. Furthermore, we observed in our studies that GO in MDA-MB-231 cells reduced viability in a dose- and time-dependent manner. When MDA-MB-231 cells were incubated with higher concentrations of GO, the cytotoxic effect on cell viability was markedly more pronounced. In contrast to MDA-MB-231 cells, the ZR-75-1 cell line was not sensitive to the GO concentrations used in our study. Shen et al. demonstrated that GO induces cytotoxicity in colorectal cancer cells through the ROS-dependent AMPK/mTOR/ULK-1 pathway [8]. Gurunathan et al. reported that GO can exert cytotoxic effects in MCF-7 cell lines [24]. Thang et al. investigated the cytotoxic effects of GO on osteosarcoma MG-63 and K_7_M_2_ cell lines, demonstrating that GO treatment reduced the viability of both osteosarcoma cell lines [23]. The cytotoxicity of GO depends on its physicochemical properties and shape. GO exhibits hydrophobic properties and disperses well in aqueous solutions. Many studies have indicated that GO is smaller and less toxic to cells than rGO [25]. The slight difference in the cytotoxic effect of GO at doses of 10 µg/mL and 300 µg/mL on MDA-MB-231 cells may be due to the reduced GO uptake by cells at the higher GO concentration range. Ou et al. hypothesized that higher concentrations of GO may cause increased formation of a protein corona that hinders the penetration of nanoparticles into MDA-MB-231 cell lines. The cytotoxic effect of GO on cancer cells can be reduced when the nanoparticles are pre-coated with FBS. Similar observations occurred when GO was coated with BSA. Notably, serum may reduce the cytotoxicity of pristine GO in J744.A1 cell lines [26]. GO can damage cell membranes by manipulating the upregulated or downregulated expression of membrane- and cytoskeleton-related genes. Additionally, F-actine filaments can be localized in GO. This effect is associated with cell cycle modifications, apoptosis, or oxidative stress [27].

Oxidative stress results from an imbalance between ROS synthesis and its inactivation and is the factor responsible for GO cytotoxicity in cells. High ROS levels in cancer cells can be induced by mitochondrial dysfunction resulting from exposure to GO nanoparticles. ROS play an important role in cellular metabolism, signaling, and homeostasis [28]. Therefore, we decided to study the influence of GO on intracellular ROS synthesis in MDA-MB-231 and ZR-75-1 cells. Our research shows that GO induced intracellular ROS generation only in MDA-MB-231 cells and not in the ZR-75-1 cell line. Preincubation of MDA-MB-231 and ZR-75-1 cells with 50 μg/mL GO + NAC resulted in a significant decrease in the intracellular ROS levels in comparison to the cells incubated with 50 μg/mL GO only. Tang et al. indicated that stimulated ROS synthesis and apoptosis were observed in K_7_M_2_ cells incubated with GO [23]. Additionally, the AMPK/mTOR signaling pathway can be activated via ROS generation. Shen et al. showed that GO induces ROS synthesis in colorectal cancer cells [8]. Upregulated ROS synthesis, exceeding a certain threshold, can induce apoptosis in mammalian cells [28].

Apoptosis can be attributed to the downregulation of the antioxidant GSH. This phenomenon may be associated with cell cycle disruptions, including G0/G1 reduction and G2/M cell cycle arrest, along with upregulation of ROS synthesis. Many studies have indicated that nanomaterials can arrest the cell cycle at various phases [29]. In our study, we observed that GO downregulated G0/G1 and G2/M cell cycle arrest and induced apoptosis in MDA-MB-231 cells. We did not observe this phenomenon in ZR-75-1 cell lines. Furthermore, to validate these cell cycle analysis results, we conducted Western immunoblot analyses of P21 protein, which is involved in inhibiting cell cycle progression. Interestingly, Bera et al. demonstrated that GO led to cell cycle arrest in the S phase, in contrast to the primary observed cell cycle disruption via a microtubule-targeting agent in mitosis (G2/M) [30]. Jiang et al. investigated the effect of Ori@GE11-GO on cell cycle progression in the KYSE-30 and EC109 esophageal cancer cell lines. Studies have shown that Ori@GE11-GO nanoparticles reduce the G0/G1 and S phases of the cell cycle and significantly increase the percentage of G2/M phase in esophageal cancer cells [31]. Interestingly, our previous study on breast cancer cell lines demonstrated that rGO could upregulate the S phase of the cell cycle and induce apoptosis/autophagy [12]. Cell cycle arrest induced by GO or rGO nanomaterials may result from the nanomaterials’ localization to F-actin filaments. For the cell cycle to progress, F-actin filaments must be intact.

Furthermore, changes in cell cycle progression may be associated with disturbances of the ERK pathway in PC12 cancer cell lines [32]. Apoptosis is the most common form of cell death and is defined as cell self-destruction [26]. Apoptosis dysfunction can lead to tumor progression or cancer cell survival [8]. In this study, we demonstrated that GO induces apoptosis and necrosis in MDA-MB-231 and mild necrosis, without apoptosis, in ZR-75-1 cells. To validate the cytometric results, we performed analysis by fluorescence microscopy, BE/OA staining and Western immunoblotting analysis. Our microscopic studies demonstrated nuclear chromatin condensation and marginalization only in MDA-MB-231 cells, along with upregulated Noxa, Cyt c, and P-Bim protein expression and downregulated Bcl-xl protein expression in these cells. Furthermore, TEM analysis indic ated results similar to those observed in fluorescence microscopy.

GO and rGO can physically damage cell membranes. Ou et al. showed that graphene nanoparticles induced apoptosis, necrosis, and inflammation in mice lungs after inhalation [26]. Shen et al. demonstrated apoptosis in colorectal cancer cells induced by GO nanoparticles [8]. In the same study, scientists showed that GO upregulated the expression of the proapoptotic Bax and cleaved the expression of caspase-3 and decreased the expression of the antiapoptotic protein Bcl-2 in colon cancer cells. In the same study, Shen et al. observed necrosis in cells incubated in the presence of GO in colorectal cancer cells [8]. Similar observations were made by Tang et al., who investigated a proapoptotic effect of GO on osteosarcoma cells [23]. Feng X et al. observed that GO induced apoptosis by impairing autophagic flux in rat pheochromocytoma PC12 cell lines [5]. The lack of apparent apoptosis in our study in ZR-75-1 cell lines may indicate the activation of cytoprotective autophagy induced by GO nanoparticles. Physical damage to the biological membrane by hydrophilic GO leads to severe permeabilization of the outer mitochondrial membrane and changes in mitochondrial membrane potential. Furthermore, GO can induce ROS generation and mitochondrial membrane potential disruption in various cancer cells. Increased ROS synthesis induced by the MAPK and TGF-β signaling pathways, as well as activated capsase-9 and caspase-3/7, promotes apoptosis via the intracellular apoptotic pathway [26]. Our study demonstrated that GO induced the activation of capsase-9 and caspase-3/7 in MDA-MB-231 cells, but not in ZR-75-1 cells, under GO conditions.

Ou et al. showed that graphene nanomaterials induced apoptosis in cancer cells by activating caspase-3. Interestingly, GO nanoribbons or GO-polyethylenimine induced apoptotic death by translocating to the nucleus [4]. Banerjee et al. indicated that GO-Au nanoparticles induced a decrease in the expression of the antiapoptotic protein Bcl-2, an increase in the expression of the protein Bax, and activation of caspase-9 and caspase-3 in breast cancer cells [33]. In our study, we demonstrated a decrease in mitochondrial membrane potential and an increase in cytochrome c release from mitochondria induced by GO in MDA-MB-231 cells but not in ZR-75-1 cells. This phenomenon was accompanied by ROS generation in MDA-MB-231 cells but not in ZR-75-1 cells incubated with GO. The MDA-MB-231 cells we observed in TEM had mitochondria with a highly condensed mitochondrial matrix, indicating a reduced mitochondrial membrane potential. Cellular mitochondria are responsible for producing significant amounts of ATP and are important targets for cellular oxidative damage [34]. Mitochondrial membrane potential is important for ATP synthesis. Depolarization of the mitochondrial membrane, induced by GO, leads to mitochondrial dysfunction and increased ROS synthesis. This process has been suggested to underlie the cytotoxic effects of GO in cancer cells [35].

Necrosis is an alternative type of cell death induced by inflammation or cellular damage by GO. GO can induce high Ca^2+^ levels in the cells, which in turn trigger the opening of the mitochondrial permeability transition pore, leading to necrosis. Macrophages incubated with GO were characterized by the induction of necrosis through the activation of TLR4 signaling and autocrine TNF-a synthesis [26]. In the present study, we observed that GO induced strong necrosis in MDA-MB-231 cells, whereas only mild necrosis was induced in ZR-75-1 cells. Similarly to apoptosis, the lower level of necrosis in ZR-75-1 cell lines may be due to the cytoprotective effect of autophagy. The authors suggest that GO may act in two ways. In MDA-MB-231 cells, it leads to programmed cell death—that is, apoptosis or necrosis—while, in ZR-75-1 cells, it may lead to autophagy.

Cytoprotective autophagy in breast cancer cells is a survival mechanism under GO-induced stress conditions. Therefore, inhibition of autophagy may be a possible therapeutic avenue for graphene oxide-resistant breast cancer. Cytoprotective autophagy helps cancer cells survive by recycling cytoplasmic materials to provide energy and essential ingredients [36]. In many cases, the induction of protective autophagy, which decreases sensitivity to chemotherapeutic drugs and radiation, is associated with drug-resistant cancer cells [37,38]. One of the key biophysical parameters of cells is their surface charge. This charge depends on the composition of the biological membrane and the physiological state of the cells. Cancer cells are more depolarized (negative Vm values) than normal cells. For this reason, graphene materials such as GO can bind to cancer cell membranes. Graphene oxide contains numerous oxygen functional groups and prefers adhesion to cancer cells over normal cells (fibroblasts).

Ion channels play a key role in regulating the functioning of normal and cancer cells and are involved in the proliferation and migration of glioblastoma multiforme cells [39]. Szczepaniak et al. demonstrated that rGO affects the expression of voltage-dependent ion channel genes, leading to changes in the membrane potential of glioblastoma multiforme cells [39]. It is believed that graphene nanomaterials—including GO—may reduce the expression of extracellular receptors and limit tumor invasiveness [39]. We think that MDA-MB-231 cells are more depolarized than ZR-75-1 cells, which are resistant to GO. For this reason, MDA-MB-231 cells undergo apoptosis during incubation with GO.

## 5. Conclusions

**Figure 8:**
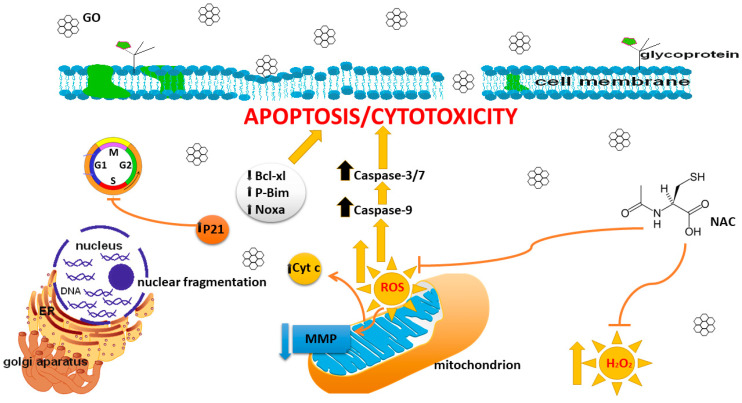
The effects of GO on apoptosis, oxidative stress, and cell cycle arrest in breast cancer MDA-MB-231 cell lines.

In summary, the in vitro results elucidated novel mechanisms underlying GO’s modulated anticancer effects. Our results indicate that GO induced cytotoxicity and oxidative stress in MDA-MB-231 but not in ZR-75-1 cell lines. The activation of caspase-9 and the decrease in mitochondrial membrane potential suggest that apoptosis was activated via the mitochondrial pathway. These results suggest the potential for future use of GO nanoparticles in the fight against breast cancer (Figure 8).

## Data Availability

The data presented in this study are available on request from the corresponding author.

## Abbreviations

AO	Acridine orange
EB	Ethidium bromide
GBMs	Graphene-based nanomaterials
GO	Graphene oxide
MTT	3-(4,5-dimethylthiazol-2-yl)-2,5-diphenyltetrazolium bromide
NAC	N-acetylcysteine
PCD	Programmed cell death
rGO	Reduced graphene oxide
ROS	Reactive oxygen species
